# Evaluating the association between caesarean delivery and weight status in early childhood in a Japanese birth cohort study

**DOI:** 10.1038/s41598-023-45316-8

**Published:** 2023-11-10

**Authors:** Chiharu Miyayama, Naho Morisaki, Kohei Ogawa, Hisako Tanaka, Hiromichi Shoji, Toshiaki Shimizu, Haruhiko Sago, Reiko Horikawa, Kevin Y. Urayama

**Affiliations:** 1 Department of Social Medicine, National Center for Child Health and Development, Tokyo, Japan; 2 Department of Pediatrics, Faculty of Medicine, Juntendo University, Tokyo, Japan; 3 Graduate School of Public Health, St. Luke’s International University, 3-6-2 Tsukiji, Chuo-Ku, Tokyo, 104-0045 Japan; 4 Center for Maternal-Fetal, Neonatal and Reproductive Medicine, National Center for Child Health and Development, Tokyo, Japan; 5 Collaborative Departments of Advanced Pediatric Medicine, Tohoku University, Miyagi, Japan; 6 Department of Endocrinology, National Center for Child Health and Development, Tokyo, Japan

**Keywords:** Epidemiology, Paediatric research

## Abstract

To examine whether the prevailing hypothesis of an association between caesarean section (CS) delivery method and increased weight status in early childhood is observed in Japanese. A total of 1277 mother-infant pairs from a prospective hospital-based mother-infant birth cohort that recruited women in their first trimester from May 2010 to November 2013 were included. We assessed the relationship between delivery method and weight status of delivered children at 1, 3 and 6 years of age. In total, 366 children (28.7%) were delivered by CS. Delivery by CS was not associated with body mass index (BMI) z-score (≥ 75 percentile) at age 1 year, (odds ratio (OR) 0.97, 95% confidence interval (CI) 0.69–1.36), 3 years (OR 0.98, 95% CI 0.67–1.42), and 6 years (OR 0.71, 95% CI 0.45–1.12), and also showed no association with low weight status (< 25th percentile). Supplemental evaluations addressing the influence of preterm births, pre-pregnancy BMI, emergency CS, and modification by breastfeeding were consistent with the primary analyses. Our findings do not support the hypothesis that children born by CS are at risk of being overweight in childhood among the Japanese population.

## Introduction

Childhood obesity is a serious public health problem worldwide, and its prevalence is increasing globally, including in Japan^1, 2^. The proportion of Japanese overweight children in 2016 was 14%, and has increased by 14% compared to 1990^3^. Childhood weight and body composition are important determinants of whether or not a person will be overweight in adulthood^4^. In addition, obesity in children is associated with many adverse health consequences, such as cardiovascular and metabolic disorders, making it more likely for young obese people to experience these later in life^5, 6^.

Caesarean delivery has attracted attention as a perinatal factor involved in the pathogenesis of childhood obesity. It has been hypothesized that because of differences in intrapartum bacterial exposure, an intestinal microbiota of infants born by caesarean section (CS) differs from that of infants born vaginally^7, 8^, and may contribute to the development of subsequent lifestyle-related diseases, such as obesity and immune-related diseases. However, the evidence is inconsistent^9–12^, which may be partly explained by differences in adjustment for confounding, the age at which the outcomes (overweight or obesity) were assessed, limitation in distinguishing between elective and non-elective CS, and/or may reflect a mechanistically complicated association that varies by variable circumstances across regions^13^.

To our knowledge, there are no studies in Japan that have directly examined the role of delivery method in affecting childhood weight trajectories and obesity. In Asia, there are reports from China showing evidence of a positive association^14–16^; but population-specificity in environmental and lifestyle circumstances makes it questionable whether the findings may be generalizable.

To address the current lack of evidence in Japanese and previous methodological constraints regarding the role of CS in influencing the weight status, we performed an analysis utilizing detailed data from a birth cohort study to investigate the association between delivery method and a child’s weight status at ages 1, 3 and 6 years. Additionally, we pursued a comprehensive examination that addressed several of the methodological issues limiting previous studies such as the confounding influence of maternal pre-pregnancy body mass index (BMI) and possible modification by postnatal breastfeeding practices in affecting the association between delivery method and child’s weight status.

## Methods

### Study population

This study utilized data from an ongoing prospective, hospital-based maternal-child birth cohort^17^ initiated in 2010 with recruitment of pregnant women who made antenatal visits to the National Center for Child Health and Development (NCCHD), a tertiary hospital in Tokyo managing approximately 2000 deliveries annually. Participants were recruited from May 2010 to November 2013 at their first antenatal visit which usually took place during weeks 6 to 14 of gestation. Written informed consent was obtained from the parents and on behalf of each participating child at two time points; first at the time of initial recruitment during pregnancy, and re-consent was requested after delivery within a few months postnatal. During the first year postnatal, follow-up was performed at 1 month, 3 months, and at 3-month intervals thereafter. From age 1 year, participants were invited for follow-up on an annual basis; some children may have missed their follow-up in certain years. We confirmed that all procedures were carried out in accordance with the principles of the Declaration of Helsinki and the Strengthening the Reporting of Observational Studies in Epidemiology (STROBE) guidelines for cohort studies. This study has been approved by the Institutional Review Board at the National Center for Child Health and Development (project number 417).

**Figure 1:**
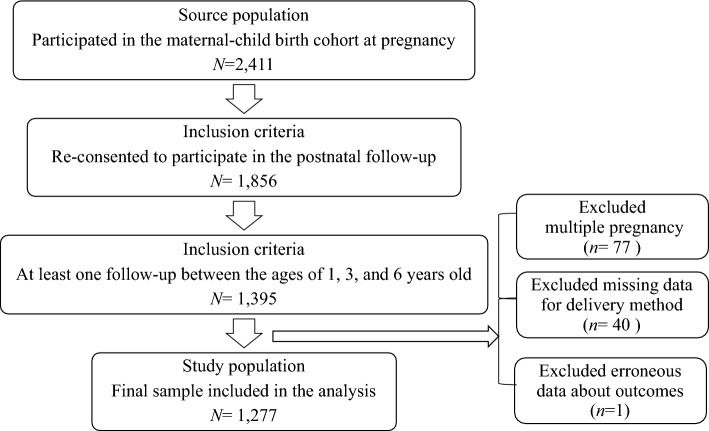
Flow chart of study population inclusion.

Of the 2411 women initially consenting at pregnancy, we limited this analysis to those who re-consented to participate in the study when the children were at the age of 1 or 3 months and who had at least one follow-up at ages 1, 3, or 6 years (Fig. 1). We excluded children who were born as part of a multiple pregnancy (n = 77) and also those missing data for the delivery method and/or erroneous data regarding the outcome (n = 41). The final number of mother–child dyads included in the analysis was 1277.

### Data collection

Data were collected through self-administered questionnaires at recruitment, including information on basic sociodemographics, pre-pregnancy characteristics, lifestyle factors, health status, pregnancy status and psychological factors. Clinical data pertaining to the pregnancy and delivery were obtained from the electronic medical records, including information on pregnancy complications, delivery characteristics, and birth outcomes. Data collection at follow-up included a combination of self-administered questionnaires and requests for visits to the hospital for in-person anthropometric measurements by medical staff and biospecimen collection which were used for other investigations. The exposure variable of primary interest was delivery method (vaginal or caesarean), and the primary outcome was child’s weight status at follow-up. Breastfeeding status was examined as a potential effect modifier since it is known to influence weight trajectories in children, as well as the gut microbiota which may be involved mechanistically^18, 19^.

### Measures of weight status

Body mass index (BMI) and age- and sex-specific percentage of overweight (POW) were used as indices to evaluate weight status at ages 1, 3, and 6 year. The BMI z-score based on age- and sex-specific population standards were calculated using the Excel-based Clinical Tools for Growth Evaluation of Children sourced from the Japanese Society for Pediatric Endocrinology^20^. This calculation tool was created based on the Infant Physical Growth Survey Report published by the Ministry of Health, Labor and Welfare in 2000^21, 22^.

POW is a percentage of how far the measured weight is from the standard weight (calculated as [measured weight – standard weight]/standard weight × 100). It is not influenced by height and is a highly useful index for comparing time-dependent changes, but it is a measure used only in Japan. Thus, we used both POW and the BMI z-score measures to allow for corroboration. Weight status was the outcome in the primary analysis, in which values based on the BMI z-score and POW distributions of this study population were used to divide into 25th percentile or less (lower weight status), 25-75th percentile (reference), and 75th percentile or more (higher weight status). Overweight in childhood is defined as BMI z-score >  + 1 SD according to the World Health Organization criteria^23^. Regarding POW, overweight is defined as ≧15% at ages 1 and 3 years and ≧20% at age 6 years. However, the prevalence of overweight at age 6 years in this study was 7.0% and 2.9% based on BMI z-score and POW, respectively^24^. Since we expected the prevalence of overweight in the current study population to be lower than the general population which affects statistical power, a cut-off at the 75th percentile value based on the BMI z-score and POW distributions of this study population was used for the primary analysis.

### Covariates

Covariates of interest for this current analysis included maternal age (29 or less, 30–34, 35–39, 40 or more years), parental education (high school or less, community college or training school, university), annual household income (less than 4 million yen, 4–8 million yen, more than 8 million yen), smoking status during pregnancy (yes or no), gestational diabetes (yes or no), primiparity (yes or no), infant’s sex (girl or boy), gestational age at birth (less than 37 weeks, 37 weeks or more), and birth weight (less than 2500g, 2500g or more). Gestational weight gain was calculated as the difference between self-reported pre-pregnancy weight and the measured weight at delivery obtained from the medical records. Maternal pre-pregnancy BMI was calculated based on self-reported weight and height and categorized as less than 18.5, 18.5–25.0, or more than 25.0. Breastfeeding status at ages 1 month and 3 months was collected by self-administered questionnaire and categorized as exclusive breastfeeding, partial breastfeeding, or no breastfeeding.

### Statistical analysis

We performed bivariate analysis by delivery method (vaginal vs caesarean) using the 25th and 75th percentile value cut-points for BMI z-score and POW in our study population as indicators of relatively lower and higher weight status, respectively.

We conducted multivariable analyses using multinomial logistic regression and calculated odds ratios (OR) and 95% confidence intervals (CI) considering three nested models; 1) the unadjusted association between delivery method and child’s BMI z-score and POW category, 2) a model adjusting for sociodemographic variables that were deemed potential confounders based on its association with delivery method (i.e. maternal age and education level), and 3) the full model additionally adjusting for potential confounding by pre-pregnancy BMI, gestational age, and birth weight. We also conducted a linear regression analysis to examine BMI z-score and POW as continuous outcomes.

To examine factors potentially influencing the results, we pursued a series supplementary evaluations and sensitivity analyses based on the full model. The association with delivery method was examined excluding emergency CS. For sensitivity analyses, children who were preterm (under 37 weeks of gestation) or had mothers with pre-pregnancy BMI greater than 25.0 (defined as overweight) were excluded. Stratified analyses were performed for exclusive breastfeeding and no exclusive breastfeeding at ages 1 and 3 months. Finally, we used logistic regression adjusting for the full set of potential confounders to examine whether delivery method was associated with an increase in BMI (yes/no) between the ages of 1 and 3 years to assess the role of adiposity rebound (AR), a phenomenon represented by changes in BMI from decreasing to increasing in early childhood. All analyses were performed using STATA SE 16.0 (STATA Corp, College Station, TX, USA). Two-sided P values of less than 0.05 were considered to be statistically significant.

## Results

Maternal and child characteristics by delivery method are shown in Table 1. Overall, the median maternal age was 37 years (range: 21 -48 years), 62.1% had a university education, and 58.2% had an annual household income of over 8 million. The median gestational age at birth was 39.3 weeks, the average birth weight was 2991.7 g, and exclusive breastfeeding after birth was 49.5% at 1 month and 56.0% at 3 months old. Of the 1277 women, 28.7% delivered by CS. Compared to women who delivered vaginally, those who delivered by CS were generally older, had higher pre-pregnancy BMI, and higher occurrence of gestational diabetes. Regarding the characteristics of children, those born by CS were more likely to be preterm and of low birth weight, and also less likely to be exclusively breastfed at both 1 and 3 months old compared with those born vaginally.

**Table 1:** Characteristics of the mothers and children by delivery method.

	Overall (n = 1277)		Vaginal delivery (n = 911)		Caesarean delivery (n = 366)		*p*
	n	(%)	n	(%)	n	(%)	
**Maternal characteristics**							
Maternal age (years)							< .001
≦29	91	(7.1)	75	(8.2)	16	(4.4)	
30–34	323	(25.3)	252	(27.7)	71	(19.4)	
35–39	593	(46.4)	419	(46.0)	174	(47.5)	
≧40	270	(21.1)	165	(18.1)	105	(28.7)	
Median (range)	37	(21–48)	36	(21–45)	38	(22–48)	
Highest education							
High school or less	80	(6.6)	50	(5.7)	30	(8.6)	.136
Community college, training school	382	(31.3)	281	(32.3)	101	(28.9)	
University	758	(62.1)	540	(62.0)	218	(62.5)	
Annual income (yen)							
< 4 million	89	(7.6)	55	(6.6)	34	(10.2)	.069
4–8 million	400	(34.2)	296	(35.4)	104	(31.1)	
> 8 million	681	(58.2)	485	(58.0)	196	(58.7)	
Pre-pregnancy BMI (kg/m^2^)							
< 18.5	249	(19.7)	188	(20.8)	61	(17.0)	.010
18.5–25	947	(75.0)	678	(75.0)	269	(74.9)	
≧25	67	(5.3)	38	(4.2)	29	(8.1)	
Median (range)	20.1	(15.0–37.3)	20.0	(15.0–36.1)	20.5	(15.2–37.3)	
Gestational weight gain (kg)							
Mean (SD)	9.8	(3.5)	9.9	(3.4)	9.6	(3.8)	.243
Gestational diabetes							
No	1214	(95.1)	873	(95.8)	341	(93.2)	.047
Yes	63	(4.9)	38	(4.2)	25	(6.8)	
Smoking during pregnancy							
No	1248	(97.8)	892	(97.9)	356	(97.5)	.675
Yes	28	(2.2)	19	(2.1)	9	(2.5)	
Primiparity							
No	465	(36.4)	320	(35.1)	145	(39.6)	.132
Yes	812	(63.6)	591	(64.9)	221	(60.4)	
**Child characteristics**							
Gender							
Boy	648	(50.7)	456	(50.0)	192	(52.5)	.437
Girl	629	(49.3)	455	(50.0)	174	(47.5)	
Gestational age (week)							
Preterm (< 37)	67	(5.2)	29	(3.2)	38	(10.4)	< .001
Full-term (≧37)	1210	(94.8)	882	(96.8)	328	(89.6)	
Median (range)	39.3	(23.9–41.9)	39.6	(32.2–41.7)	38.4	(23.9–41.9)	
Birth weight (gram)							
Mean (SD)	2992	(429.5)	3023	(386.8)	2914	(513.0)	
< 2500	128	(10.0)	72	(7.9)	56	(15.3)	< .001
≧2500	1149	(90.0)	839	(92.1)	310	(84.7)	
Breastfeeding at 1 month old							
Exclusive	588	(49.5)	447	(53.0)	141	(41.1)	.001
Partial	579	(48.8)	384	(45.5)	195	(56.9)	
None	20	(1.7)	13	(1.5)	7	(2.0)	
Breastfeeding at 3 months old							
Exclusive	597	(56.0)	448	(58.2)	149	(50.3)	.037
Partial	442	(41.5)	306	(39.7)	136	(46.0)	
None	27	(2.5)	16	(2.1)	11	(3.7)	

**Figure 2:**
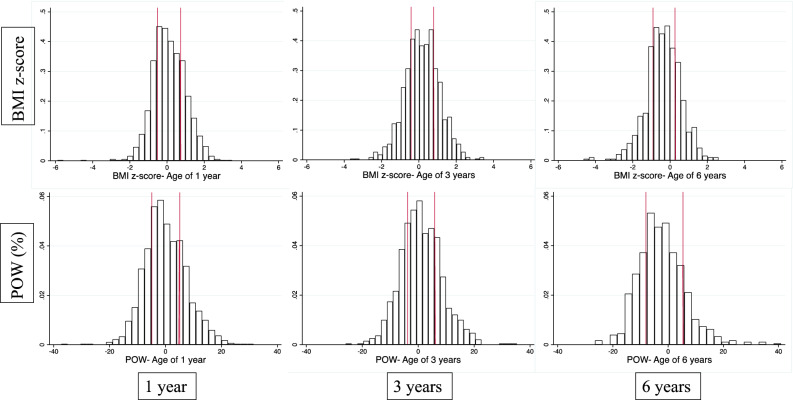
Distribution for BMI z-score and POW at the ages 1, 3, and 6 years. *Note.* Red line represents cutoff points at the 25th and 75th percentile.

The distribution of both BMI z -score and POW in this study population tended to be close to normal at ages 1 and 3 years, but appeared to be slightly skewed towards lower values at age 6 years compared to the Japanese standard population (Fig. 2). The results of the BMI z-score and POW percentile categories by delivery method in bivariate analyses showed no marked associations at all 3 follow-up ages (Table 2). Using clinically defined cut-points for overweight also showed similar distributions between caesarean and vaginal deliveries (Supplementary Table S1).

**Table 2:** Evaluation of delivery method and weight status measures (BMI z-score and POW) at 1, 3, and 6 years of age.

	Vaginal delivery		Caesarean delivery		*p*
	n	(%)	n	(%)	
**Follow-up at 1 year old (n = 1176)**					
BMI z-scores					
< 25%tile	211	(25.1)	83	(24.9)	.931
25–75%tile	423	(50.2)	165	(49.4)	
> 75%tile	208	(24.7)	86	(25.8)	
Mean (SD):	0.10	(0.92)	0.15	(0.87)	
POW (%)					
< 25%tile	209	(24.8)	85	(25.5)	.798
25–75%tile	426	(50.6)	162	(48.5)	
> 75%tile	207	(24.6)	87	(26.1)	
Mean (SD):	0.11	(7.67)	0.04	(7.48)	
**Follow-up at 3 years old (n = 895)**					
BMI z-scores					
< 25%tile	151	(24.2)	72	(26.5)	.711
25–75%tile	313	(50.2)	136	(50.0)	
> 75%tile	159	(25.5)	64	(23.5)	
Mean (SD):	0.18	(0.93)	0.17	(0.97)	
POW (%)					
< 25%tile	158	(25.4)	68	(25.0)	.941
25–75%tile	311	(49.9)	138	(50.7)	
> 75%tile	154	(24.7)	66	(24.3)	
Mean (SD):	1.18	(7.55)	1.07	(7.65)	
**Follow-up at 6 years old (n = 647)**					
BMI z-scores					
< 25%tile	119	(26.0)	42	(22.2)	.421
25–75%tile	222	(48.5)	102	(54.0)	
> 75%tile	117	(25.6)	45	(23.8)	
Mean (SD):	-0.35	(0.97)	-0.37	(0.87)	
POW (%)					
< 25%tile	113	(24.7)	49	(25.9)	.797
25–75%tile	227	(49.6)	96	(50.8)	
> 75%tile	118	(25.8)	44	(23.3)	
Mean (SD):	-2.27	(8.60)	-2.30	(8.53)	

In the fully adjusted multinomial logistic regression analysis accounting for sociodemographic and perinatal variables, CS was not associated with higher weight status (≥75th percentile: OR 0.97, 95% CI, 0.69–1.36) or lower weight status (<25th percentile: OR 0.94, 95% CI, 0.67–1.31) at age 1 year compared to middle weight status (25-75th percentile) (Table 3). The results did not dramatically change at ages 3 years and 6 years for higher and lower weight status when compared to middle weight status, with risk estimates of about 1.00 or below. The strongest associated factor was maternal pre-pregnancy BMI, in which a BMI of greater than 25 was associated with an increased risk of higher weight status of the child at age 1 year (OR 4.03, 95% CI 2.02–8.03), 3 years (OR 2.51, 95% CI 1.17–5.42), and 6 years (OR 7.94, 95% CI 2.77–22.79). When POW was examined in place of BMI z-score, similar results were observed at ages 1 year, 3 years, and 6 years (Supplementary Table S2). Analysis of BMI and POW as continuous variables in a linear regression was also performed, and results were consistent with a finding of no association with delivery method at all three follow-up ages (data not shown).

**Table 3:** Multivariable multinomial logistic regression results for the association between delivery method and weight status based on BMI z-score percentiles at age 1 year, 3 years, and 6 years.

(comparison: 25th -75th percentile)	BMI z-score at age 1 year				BMI z-score at age 3 years				BMI z-score at age 6 years			
	< 25th percentile		≥ 75th percentile		< 25th percentile		≥ 75th percentile		< 25th percentile		≥ 75th percentile	
	OR^a^	(95% CI)	OR^a^	(95% CI)	OR^a^	(95% CI)	OR^a^	(95% CI)	OR^a^	(95% CI)	OR^a^	(95% CI)
Delivery method (vs. vaginal)												
Cesarean section	0.94	(0.67–1.31)	0.97	(0.69–1.36)	0.90	(0.62–1.32)	0.98	(0.67–1.42)	0.85	(0.54–1.34)	0.71	(0.45–1.12)
Maternal age (vs. < 30)												
30–34	1.01	(0.55–1.86)	1.08	(0.56–2.09)	1.35	(0.66–2.78)	2.89	(0.95–8.75)	0.75	(0.31–1.83)	1.10	(0.36–3.37)
35–39	1.09	(0.60–1.95)	1.04	(0.55–1.96)	1.09	(0.54–2.19)	3.88	(1.32–11.40)	0.68	(0.28–1.61)	1.31	(0.44–3.89)
≥ 40	1.27	(0.68–2.38)	1.10	(0.56–2.18)	1.49	(0.72–3.12)	4.24	(1.40–12.84)	0.69	(0.28–1.69)	1.05	(0.34–3.25)
Maternal education (vs. high school or less)												
Community college	0.53	(0.30–0.94)	1.20	(0.60–2.42)	0.80	(0.40–1.60)	1.13	(0.49–2.62)	0.63	(0.27–1.43)	1.31	(0.47–3.63)
University	0.54	(0.32–0.93)	1.48	(0.75–2.90)	0.59	(0.30–1.16)	1.37	(0.61–3.08)	0.44	(0.20–0.97)	1.04	(0.38–2.81)
Pre-pregnancy BMI (vs. < 18.5)												
18.5–25	0.67	(0.47–0.94)	1.95	(1.24–3.05)	0.81	(0.54–1.22)	1.53	(0.95–2.46)	0.54	(0.34–0.86)	2.26	(1.21–4.25)
> 25	0.43	(0.19–0.98)	4.03	(2.02–8.03)	0.52	(0.21–1.28)	2.51	(1.17–5.42)	0.67	(0.19–2.40)	7.94	(2.77–22.79)
Gestational age (vs. < 37 weeks)												
≥ 37 weeks	0.85	(0.40–1.81)	0.55	(0.25–1.21)	1.18	(0.48–2.87)	0.47	(0.20–1.11)	2.11	(0.54–8.19)	0.48	(0.18–1.27)
Birth weight (vs. 2500–4000)												
< 2500	1.22	(0.74–2.01)	0.36	(0.18–0.72)	1.98	(1.11–3.52)	0.40	(0.18–0.89)	1.36	(0.67–2.74)	0.77	(0.35–1.67)
> 4000	0.41	(0.05–3.51)	1.59	(0.42–6.07)	-	-	1.97	(0.38–10.14)	0.62	(0.06–6.17)	1.44	(0.23–9.00)

BMI = body mass index; OR = odds ratio; CI = confidence interval.

^a^Odds ratio (OR) and 95% confidence interval (CI) was calculated using multinomial logistic regression including the variables shown in the table.

A series of supplemental evaluations and sensitivity analyses were performed to confirm the lack of association between delivery method and weight status categories (Table 4). Sensitivity analyses excluding preterm births, children of mothers with pre-pregnancy BMI greater than 25.0, and emergency CS, showed risk estimates close to null or below 1.0, similar to the main analysis, for both BMI z-score and POW. In analyses stratified by breastfeeding status at 1 month old, there was no significant association between delivery method and BMI z-score at age 1 year, 3 years or 6 years. Finally, a multivariable evaluation of the association between delivery method and its influence on early adiposity rebound (changes in BMI between ages 1 and 3 years) showed no marked association (data not shown).

**Table 4:** Summary of results of supplementary evaluations and sensitivity analysis examining the association between cesarean delivery and BMI z-score and POW of 75th percentile or greater (higher weight status) compared to 25th-50th percentile.

	BMI z-score		POW	
	OR	95% CI^a^	OR	95% CI^a^
**Follow-up at age 1 year**				
Main analysis results (all children)	0.97	(0.69–1.36)	1.02	(0.73–1.43)
Excluded preterm births	1.01	(0.71–1.42]	1.08	(0.77–1.52)
Excluded pre-pregnancy BMI ≥ 25.0	1.03	(0.72–1.46)	1.05	(0.74–1.49)
Excluded emergency CS	1.17	(0.77–1.76)	1.12	(0.74–1.69)
Children exclusively breastfed	1.04	(0.60–1.83)	1.21	(0.73–2.02)
Children not exclusively breastfed	0.99	(0.63–1.56)	1.09	(0.70–1.70)
**Follow-up at age 3 years**				
Main analysis results (all children)	0.98	(0.67–1.42)	0.88	(0.61–1.29)
Excluded preterm births	1.00	(0.68–1.48)	0.90	(0.61–1.33)
Excluded pre-pregnancy BMI ≥ 25.0	0.96	(0.65–1.42)	0.90	(0.60–1.33)
Excluded emergency CS	1.03	(0.66–1.62)	0.95	(0.60–1.50)
Children exclusively breastfed	1.26	(0.69–2.30)	1.20	(0.65–2.24)
Children not exclusively breastfed	0.83	(0.50–1.39)	0.72	(0.43–1.20)
**Follow-up at age 6 years**				
Main analysis results (all children)	0.71	(0.45–1.12)	0.94	(0.60–1.47)
Excluded preterm births	0.75	(0.47–1.20)	0.99	(0.63–1.58)
Excluded pre-pregnancy BMI ≥ 25.0	0.79	(0.49–1.26)	0.97	(0.61–1.53)
Excluded emergency CS	0.70	(0.40–1.25)	0.95	(0.54–1.67)
Children exclusively breastfed	0.67	(0.32–1.41)	0.82	(0.40–1.72)
Children not exclusively breastfed	1.00	(0.53–1.86)	1.14	(0.62–2.12)

BMI = body mass index; POW = percentage of overweight; OR = odds ratio; CI = confidence interval; CS = caesarean section.

^a^Estimates were derived using multinomial logistic regression adjusting for maternal age, educational status, maternal pre-pregnancy BMI, gestational age and birth weight (Model 3).

## Discussion

After adjusting for key confounders and confirmation with a series of supplemental analyses, no association was observed between the delivery method and the child’s weight status at ages 1, 3, and 6 years. These observations are in contrast to findings of previous studies, including meta-analyses showing that children born by CS are at higher risk of developing obesity in childhood^10, 25^. However, of consideration is the fact that these meta-analyses comprised studies primarily conducted among populations from western countries, in which circumstances may be different than Japan. The current study represents one of the first thorough epidemiological evaluations of the role of delivery method in influencing weight status in children in East Asians.

One possibility for these contrasting results is that child’s weight status may be influenced by maternal BMI and eating habits which may differ across populations^26, 27^. A German study, which did not find a significant association with delivery method, described that there may be other dominating risk factors, such as dietary habits and physical activity as children grow up, influencing the risk of obesity in later life more so than the risk conferred by microbial exposures during child birth^11^. The mothers in our study population have generally lower BMI during pregnancy compared to the general pregnancy population in Japan (BMI < 18.5; 19.7% vs 11.2%, BMI≧ 25; 5.3% vs 21.9%)^28^. It may be possible that certain lifestyle characteristic of our study population may have offset the negative influences of caesarean delivery that would have otherwise contributed to the risk of overweight in other populations. Another possibility for the inconsistency may be due to differences in the distribution of microbiota among populations across different countries^29^. Thus, the association between delivery method and weight status might be different in Japan, and may not be a primary factor effecting obesity risk. However, we did not examine the profile of the infants’ gut microbiota, so we were not able to confirm this point.

Previous studies reported that breastfeeding during infancy is associated with differences in the child’s microbiome^7, 19^. Therefore, postnatal breastfeeding practices may affect the infant’s gut microbiota and modify the association between delivery method and future weight status. There is evidence that cessation of breastfeeding influences the development of adult-like microbiota more than the introduction of solid foods^30^. In our stratified evaluations specifically examining the association among exclusive breastfed and non-exclusive breastfed children, no association was observed in both populations. Preterm infants also tend to be born by CS for a variety of reasons and often receive medical care after birth. Considering that the environment is different from that of term infants, we excluded preterm infants in a sensitivity analysis, but observed no marked effect on the results.

We examined the association between delivery method and child’s weight status at three different time points, ages 1, 3, and 6 years. A Danish study reported that caesarean delivery was associated with an increased risk of obesity among men at age 18^31^. A systematic review also showed that delivery by CS was associated with an increased risk of obesity not only in early childhood (0–5 years), but also in adolescents (5–18 years), and adulthood^32^. While there may be a possibility that an association with delivery method may become apparent with longer follow-up into adolescence, we speculate that this may not be likely as there was no tendency for rising risk estimates with increasing age.

A planned CS is performed prior to labor or membrane rupture and can deprive the vaginal microbiota of exposure. In contrast, a membrane rupture may have already occurred in some cases of emergency CS. Therefore, in additional confirmatory analyses, we excluded children born by emergency CS, but results showed no notable difference. Several studies have examined the association between selective or non-selective CS and obesity in childhood, but have not obtained additional clarity for the association^33–35^.

Other mechanistic considerations are necessary to aid the interpretation of results. Delivery method may influence child’s weight trajectories by affecting the maturation of the gut microbiota, but CS is also known to affect feeding patterns due to effects on maternal hormones, and metabolism of fat and glucose. This can have long-term effects on appetite and metabolic regulation^36^. Another mechanism that may explain the association between CS and subsequent offspring obesity is through the effects of stress exposure during labor on cell maturation and DNA hypermethylation which in turn affects metabolic function^36, 37^. Given these biologically plausible mechanisms, the pathways potentially linking delivery method and child weight trajectories appear diverse and may be influenced by other factors related to the child’s environment.

The phenomenon that BMI changes from decreasing to increasing in early childhood is known as adiposity rebound, and it has been shown that the earlier it starts, the higher the risk of developing obesity and lifestyle-related diseases^38–40^. A Japanese study reported that children who showed an increase in BMI between the ages of 1.5 and 3 years, which is usually a period of a decreased or stable BMI due to changes in body composition, tended to have higher insulin resistance^41^. Other studies have concluded that early adiposity rebound was associated with factors related to metabolic syndrome, including type 2 diabetes^42^, elevated blood pressure, and dyslipidemia^39^. Evaluation of the phenomenon in our study showed no association between delivery method and early adiposity as a potential predictor of obesity risk later in life.

To the best of our knowledge, this is the first study on the association between delivery method and child’s weight status in Japan. A major strength of this study was the use of two documented measures of weight status, BMI z-score and POW. In addition, detailed data collection allowed us to address potential confounders such as pre-pregnancy BMI and effect modification by early breastfeeding status in a sample of mother–child dyads. Distribution of weight status of this Japanese population enabled us to uniquely examine the effects of delivery method on both overweight and underweight tendencies, and we were able to separate out CS into either planned or emergency by accessing detailed clinical data. These are features that only limited previous studies have been able to address.

There are also several limitations that should be acknowledged. First, there was loss to follow-up over time, which may have affected statistical power and introduced selection bias. While certain analyses may have had reduced precision due to sample size, the risk estimates for the primary analysis and the series of supplemental analyses were consistently null suggesting minimal support for the prevailing hypothesis. The single-center nature of the study population also warrants further examination in other populations. In addition, we compared baseline demographics between participants analyzed at age 6 years and those who were lost to follow-up. We confirmed that the overall distribution of characteristics, including delivery method, were similar between those included and excluded. Secondly, there was a lack of information on maternal diet during gestation and lactation and the offspring’s early life antibiotic exposure, which might be associated with the distribution of microbiota^7, 44^. A previous study reported that administration of antibiotics up to 6 months of age increased the risk of overweight in children of normal weight mothers^12^. Additionally, several studies have reported that maternal high-fat diet induces changes in microbial ecology, affecting offspring's neurodevelopment and having long-term effects on brain health and behavior as well as obesity^43, 45^. Another limitation was a lack of complete information about the father’s characteristics precluding our ability for a thorough assessment of paternal factors. A previous study has shown that paternal BMI is associated with the child’s obesity risk^46^. Finally, the physical measurements were taken by multiple medical professionals and there may have been varying degrees of measurement error. However, as a single facility study that used the same measuring instruments across the children, we do not suspect this to be a major source of bias.

Taken together, the current study represents one of the few rigorous studies conducted on this topic, particularly in Japanese, and results do not support the hypothesis of an increased risk of overweight associated with caesarean delivery. It seems likely that exposures manifested through delivery method may not be a major factor contributing to the child’s weight status; however, it is yet unknown whether there are longer-term effects that influence the overweight tendencies in adolescents and into adulthood. Such a finding suggests that there may not be an immediate concern regarding caesarean deliveries and its influence on childhood obesity, and public health efforts should focus on various prevention programs for monitoring, evaluating, and providing education about healthy lifestyle such as consumption of healthy foods and physical activity^47^. At the same time, care for women before conception and during pregnancy about healthy dietary and lifestyle factors is also important^48, 49^.

In conclusion, we found no association between delivery method and child’s weight status at age 1, 3, and 6 years after careful consideration of a broad range of methodological issues. Further studies are needed considering longer observation periods and target population characteristics to confirm our findings and explore the mechanisms for the potential association.

### Supplementary Information

Supplementary Information.

## Data Availability

Data used in this study may be available on request from the corresponding author.
