# Supplementary material for: Evaluating the association between caesarean delivery and weight status in early childhood in a Japanese birth cohort study
Source: Sci Rep. 2023 Nov 10;13:19612. doi: 10.1038/s41598-023-45316-8 (PMC10638261; doi:10.1038/s41598-023-45316-8)
Supplement: Supplementary file 1 — Supplementary Information. [file 41598_2023_45316_MOESM1_ESM.docx]

**Supplementary Table 1.** Bivariate evaluation using the clinical criteria for overweight in Japan

|  | Overall | | Vaginal | | CS | | p ^a^ |
| --- | --- | --- | --- | --- | --- | --- | --- |
| *N* (%) | 1277 | (100) | 911 | (71.3) | 366 | (28.7) |  |
| BMI z-scores at 1 year old (SD) |  |  |  |  |  |  |  |
| ≦1.0 | 989 | (84.1) | 710 | (84.3) | 279 | (83.5) | .738 |
| >1.0 | 187 | (15.9) | 132 | (15.7) | 55 | (16.5) |  |
| Missing | 101 |  |  |  |  |  |  |
| POW at 1 year old (%) |  |  |  |  |  |  |  |
| <15.0 | 1135 | (96.5) | 810 | (96.2) | 325 | (97.3) | .351 |
| ≧15.0 | 41 | (3.5) | 32 | (3.8) | 9 | (2.7) |  |
| Missing | 101 |  |  |  |  |  |  |
| BMI z-scores at 3 years old (SD) |  |  |  |  |  |  |  |
| ≦1.0 | 735 | (82.1) | 516 | (82.8) | 219 | (80.5) | .407 |
| >1.0 | 160 | (17.9) | 107 | (17.2) | 53 | (19.5) |  |
| Missing | 382 |  |  |  |  |  |  |
| POW at 3 years old (%) |  |  |  |  |  |  |  |
| <15.0 | 858 | (95.9) | 597 | (95.8) | 261 | (96.0) | .929 |
| ≧15.0 | 37 | (4.1) | 26 | (4.2) | 11 | (4.0) |  |
| Missing | 382 |  |  |  |  |  |  |
| BMI z-scores at 6 years old (SD) |  |  |  |  |  |  |  |
| ≦1.0 | 602 | (93.0) | 423 | (92.4) | 179 | (94.7) | .285 |
| >1.0 | 45 | (7.0) | 35 | (7.6) | 10 | (5.3) |  |
| Missing | 630 |  |  |  |  |  |  |
| POW at 6 years old (%) |  |  |  |  |  |  |  |
| <20.0 | 628 | (97.1) | 442 | (96.5) | 186 | (98.4) | .192 |
| ≧20.0 | 19 | (2.9) | 16 | (3.5) | 3 | (1.6) |  |
| Missing | 630 |  |  |  |  |  |  |

*Note.* CS= caesarean section; BMI= body mass index; POW= percentage of overweight

^a^ p-values were derived based on chi-squared tests evaluating the relationship between delivery method and clinically-defined overweight status as BMI z-score > +1 SD according　to the World Health Organization criteria; for POW, it is defined as ≧15% at ages 1 and 3　years and ≧ 20% at age 6 years.

**Supplementary Table 2.** Multivariable adjusted multinomial logistic regression results for the association between delivery method and POW categories at age 1 year, 3 years, and 6 years

|  | POW at age 1 year | | | | POW at age 3 years | | | | POW at age 6 years | | | |
| --- | --- | --- | --- | --- | --- | --- | --- | --- | --- | --- | --- | --- |
| (comparison: 25^th^ -75^th^ percentile) | <25^th^ percentile | | ≥75^th^ percentile | | <25^th^ percentile | | ≥75^th^ percentile | | <25^th^ percentile | | ≥75^th^ percentile | |
|  | OR ^a^ | (95% CI) | OR ^a^ | (95% CI) | OR ^a^ | (95% CI) | OR ^a^ | (95% CI) | OR ^a^ | (95% CI) | OR ^a^ | (95% CI) |
| Delivery method (vs. vaginal) | | |  |  |  |  |  |  |  |  |  |  |
| Cesarean section | 1.01 | (0.73-1.42) | 1.02 | (0.73-1.43) | 0.93 | (0.64-1.36) | 0.88 | (0.61-1.29) | 0.84 | (0.53-1.33) | 0.94 | (0.60-1.47) |
| Maternal age (vs. <30) |  |  |  |  |  |  |  |  |  |  |  |  |
| 30-34 | 1.39 | (0.75-2.58) | 1.50 | (0.76-2.96) | 1.19 | (0.57-2.47) | 1.61 | (0.61-4.21) | 0.48 | (0.19-1.20) | 0.54 | (0.19-1.54) |
| 35-39 | 1.26 | (0.69-2.28) | 1.40 | (0.73-2.68) | 1.05 | (0.52-2.13) | 2.47 | (0.98-6.22) | 0.53 | (0.22-1.30) | 0.79 | (0.29-2.15) |
| ≥40 | 1.25 | (0.67-2.37) | 1.24 | (0.62-2.49) | 1.41 | (0.67-2.96) | 2.47 | (0.95-6.46) | 0.62 | (0.25-1.56) | 0.64 | (0.22-1.82) |
| Maternal education (vs. high school or less) | | |  |  |  |  |  |  |  |  |  |  |
| Community college | 0.40 | (0.23-0.72) | 0.75 | (0.38-1.47) | 0.67 | (0.34-1.34) | 1.23 | (0.51-2.96) | 0.54 | (0.24-1.23) | 1.19 | (0.43-3.35) |
| University | 0.38 | (0.22-0.65) | 0.97 | (0.50-1.85) | 0.49 | (0.25-0.95) | 1.41 | (0.60-3.28) | 0.37 | (0.17-0.82) | 1.03 | (0.38-2.81) |
| Pre-pregnancy BMI (vs. <18.5) | | |  |  |  |  |  |  |  |  |  |  |
| 18.5-25 | 0.64 | (0.45-0.92) | 1.86 | (1.19-2.89) | 0.81 | (0.54-1.22) | 1.71 | (1.05-2.77) | 0.63 | (0.39-1.00) | 1.90 | (1.05-3.44) |
| >25 | 0.53 | (0.24-1.17) | 4.10 | (2.05-8.21) | 0.49 | (0.20-1.18) | 2.36 | (1.08-5.15) | 2.73 | (0.74-10.05) | 14.54 | (4.23-49.98) |
| Gestational age (vs. <37 weeks) | | |  |  |  |  |  |  |  |  |  |  |
| ≥37 weeks | 1.63 | (0.75-3.56) | 0.84 | (0.38-1.86) | 1.12 | (0.46-2.72) | 0.44 | (0.19-1.03) | 0.59 | (0.18-1.95) | 0.35 | •0.13-0.96) |
| Birth weight (vs. 2500-4000) | |  |  |  |  |  |  |  |  |  |  |  |
| <2500 | 2.01 | (1.22-3.29) | 0.43 | (0.21-0.87) | 1.86 | (1.04-3.30) | 0.39 | (0.17-0.89) | 0.55 | (0.24-1.23) | 0.68 | (0.32-1.43) |
| >4000 | 0.56 | (0.06-5.08) | 2.64 | (0.69-10.05) | 0 | (0-.) | 2.00 | (0.39-10.36) | 1.98 | (0.27-14.49) | 2.28 | (0.31-16.87) |
|  |  |  |  |  |  |  |  |  |  |  |  |  |

*Note.* POW= percentage of overweight; OR= odds ratio; CI= confidence interval

^a^ Odds ratio (OR) and 95% confidence interval (CI) was calculated using multinomial logistic regression including the variables shown in the table.
